# Peripheral longitudinal consolidations

**DOI:** 10.36416/1806-3756/e20250354

**Published:** 2025-11-19

**Authors:** Edson Marchiori, Bruno Hochhegger, Gláucia Zanetti

**Affiliations:** 1. Universidade Federal do Rio de Janeiro, Rio de Janeiro (RJ) Brasil.; 2. University of Florida, Gainesville (FL) USA.

**Figure 1:**
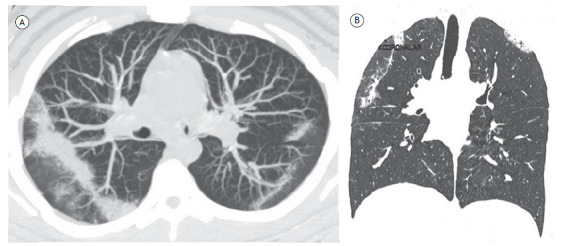
In A, axial chest CT scan showing band-like pulmonary consolidations, longitudinal to the pleural surface, in the upper lobes, predominantly on the right. In B, coronal reconstruction demonstrating the extent of the consolidations.

A 21-year-old man complained of a dry cough, progressive dyspnea, and fever for two months. He had peripheral eosinophilia of 1,800/mm^3^ (27%). A CT scan showed peripheral longitudinal bands of consolidation in the upper lobes (Figure 1). The final diagnosis was chronic eosinophilic pneumonia (CEP).

Peripheral consolidations can occasionally occur in some infections (H1N1, COVID-19, leptospirosis, etc.), non-thrombotic embolisms (fat, silicone), and sarcoidosis, among others. However, in these conditions, the clinical and laboratory findings are usually sufficient for diagnosis. The main differential diagnoses in our case, once these conditions were ruled out, would be organizing pneumonia and CEP. The main aspect for the differential diagnosis was the presence of significant eosinophilia in peripheral blood. 

Eosinophilic lung diseases are a diverse group of diseases characterized by lung opacities associated with tissue or peripheral eosinophilia. Diagnosis of eosinophilic lung disease can be made if any of the following findings are present: (a) lung opacities with peripheral eosinophilia, (b) tissue eosinophilia confirmed by open lung biopsy or transbronchial biopsy, or (c) increased eosinophils in BALF. Eosinophilic lung diseases are generally classified as those of unknown cause (simple pulmonary eosinophilia, acute eosinophilic pneumonia, CEP, idiopathic hypereosinophilic syndrome) and those of known causes (allergic bronchopulmonary aspergillosis, bronchocentric granulomatosis, parasitic infection, drug reaction), as well as eosinophilic vasculitis (allergic angiitis, granulomatosis).^1^ ^,^ ^2^

Most patients with CEP have a history of asthma or atopy. It is characterized by respiratory symptoms for 2 to 4 weeks, diffuse pulmonary alveolar consolidation with air bronchogram and/or ground-glass opacities, eosinophilia in BALF (≥ 40% eosinophils) or eosinophilia in peripheral blood (≥ 1,000/mm³), and absence of other known causes of eosinophilic pneumonia. Symptoms may persist for more than one month and include cough, fever, night sweats, progressive dyspnea, malaise, and weight loss. Blood eosinophilia is present in 90% of patients, and sputum eosinophilia in 50%. Imaging studies may show linear, band-like opacities parallel to the pleural surface. The combination of this imaging finding, the presence of eosinophilia, and response to steroid treatment are usually sufficient for diagnosis, obviating the need for lung biopsy.^1^ ^,^ ^2^

The disease responds very well to steroids, and the opacities resolve within 7 to 10 days after starting corticosteroid therapy, although recurrence rate is high.
